# Severe birth injuries in neonates and associated risk factors for injury in mothers with different types of diabetes in Finland

**DOI:** 10.1002/ijgo.14073

**Published:** 2022-01-07

**Authors:** Maiju Kekki, Kati Tihtonen, Anne Salonen, Topias Koukkula, Mika Gissler, Hannele Laivuori, Tuomas T. Huttunen

**Affiliations:** ^1^ Department of Obstetrics and Gynecology Tampere University Hospital Tampere Finland; ^2^ Center for Child, Adolescent and Maternal Health Research Faculty of Medicine and Health Technology Tampere University Tampere Finland; ^3^ Faculty of Medicine and Health Technology Tampere University Tampere Finland; ^4^ Department of Pediatric and Adolescent Surgery Tampere University Hospital Tampere Finland; ^5^ Department of Orthopaedics and Traumatology Tampere University Hospital Tampere Finland; ^6^ Information Services Department THL Finnish Institute for Health and Welfare Helsinki Finland; ^7^ Department of Molecular Medicine and Surgery Karolinska Institute and Region Stockholm Academic Primary Health Care Centre Stockholm Sweden; ^8^ Institute for Molecular Medicine Finland Helsinki Institute of Life Science University of Helsinki Helsinki Finland; ^9^ Tampere University Heart Hospital Tampere University Hospital Tampere Finland

**Keywords:** brachial plexus injury, gestational diabetes, pregestational diabetes, severe birth injury, vaginal delivery

## Abstract

**Objective:**

To examine severe birth‐related injuries in neonates among mothers with different types of diabetes.

**Methods:**

Retrospective cohort study based on Finnish Medical Birth Register data from 2004 to 2017. The study included singleton neonates born vaginally with cephalic presentation (*n *= 623 649) after 35^+0^ weeks of gestation. The primary outcome variable was severe birth injury. Incidences, crude and adjusted odds ratios, and probabilities in regression analysis were calculated for different types of diabetes.

**Results:**

There were 1952/623 649 (0.3%) severe birth injuries of which brachial plexus injury occurred most frequently. The injury incidence was highest in neonates of women with type 1 or type 2 diabetes, 42/1659 (2.5%) and 10/548 (1.8%), respectively. For gestational diabetes, the injury incidence was comparable to non‐diabetic women: 422/77 810 (0.5%) and 1478/543 632 (0.3%), respectively. Shoulder dystocia, high birthweight, and vacuum‐assisted delivery were associated with the highest probability for injury. Birthweight and obesity had a stronger impact on injury risk in women with pregestational diabetes compared to other pregnancies.

**Conclusion:**

Neonates of women with pregestational diabetes have a higher risk for severe birth injury than other neonates. The injury risk in neonates delivered by women with gestational diabetes or non‐diabetic women is generally low.

## 1 INTRODUCTION

In Finland, approximately 1.7% of live‐born neonates are diagnosed with a birth injury.^1^ Most of the injuries are transient, but severe injuries can cause permanent disability and have lifelong consequences. The incidence of severe birth injury, including cranial hemorrhage, central nervous system injury, skeletal or visceral injury, and brachial plexus palsy (BPP), is reported to be between 0.2% and 0.5% in live births, and is mainly associated with vaginal deliveries.^2^, ^3^ Since the incidence of birth injuries is low and a remarkable number of cases in the general population are unpredictable, it is important to explore the risk factors and incidences associated with high‐risk pregnancies.

Maternal diabetes is a risk factor for adverse perinatal outcomes.^3^, ^4^, ^5^, ^6^, ^7^ It increases the risk for macrosomia^4^, ^8^ and shoulder dystocia (ShD),^6^, ^8^, ^9^, ^10^ which are both known risk factors for birth injury.^6^, ^11^ Maternal obesity, especially associated with gestational diabetes mellitus (GDM) and type 2 diabetes (T2D), is another risk factor for birth‐related injuries.^7^, ^12^ In Finland, the incidence of type 1 diabetes (T1D) is among the highest in the world,^13^ and the global incidence of T2D and GDM is increasing.^3^, ^12^, ^14^, ^15^ Furthermore, after the implementation of comprehensive screening, which replaced the former risk‐based screening in 2008, the prevalence of GDM in Finland has also increased.^16^ In addition, risk factors associated with birth injuries, such as obesity, ShD, and vacuum‐assisted deliveries (VAD), have increased among women with diabetes.^9^, ^16^

This study addresses severe birth injuries in vaginal deliveries after 35^+0^ weeks of gestation in women diagnosed with T1D, T2D, or GDM and compares the results to non‐diabetic pregnancies. The study aims to describe the type of injuries, calculate the incidence rates, and determine the risk factors for severe injuries in a nationwide birth cohort study.

## 2 MATERIALS AND METHODS

This nationwide population‐based cohort study was conducted using data from the Finnish Medical Birth Register (MBR) and the Care Register for Health Care (CRHC), which are maintained by the Finnish Institute for Health and Welfare. The MBR includes data on all deliveries in Finland. The MBR comprises information on the health of the mothers and neonates, interventions needed during pregnancy, delivery, and the first 7 days after birth. The data are completed by information obtained from the Central Population Register and the Cause‐of‐Death Register. The CRHC contains information on patient characteristics, diagnoses, and operations performed during the hospital stay. The coverage and accuracy of these registers have been shown to be excellent.^17^, ^18^

The study was based on register data from the years 2004 to 2017. Gestational age was limited to between 35^+0^ and 42^+6^, as birth injuries were infrequent before 35 weeks of gestation. After excluding those neonates delivered by forceps (*n *= 273, 0.03%) or those with major congenital anomalies (*n *= 18 854, 2.4%), 623 649 singleton live born neonates born vaginally with cephalic presentation were included. The outcome variables were severe birth‐related injuries coded with the Finnish implementation of the 10th Revision of International Statistical Classification of Diseases and Related Health Problems (ICD‐10) codes. The ICD‐10 codes for birth injuries detected at 0–6 days, information on the type of mothers’ diabetes, mode of delivery, and baseline characteristics were collected from the MBR. Moreover, hospital visits related to any severe birth injury diagnosis recorded in the CRHC during the first year after birth were included to increase the coverage.

Diagnosis of T1D and T2D were based on ICD‐10 codes gathered from the MBR (O24.0, E10*, and O24.1, E11*), and GDM was defined as pathologic 2‐h 75‐g oral glucose tolerance test with at least one elevated plasma glucose value determined as ≥5.3 mmol/L (95.4 mg/dl) (fasting), ≥10.0 mmol/L (180.0 mg/dl) (1 h), and ≥8.6 mmol/L (154.8 mg/dl) (2 h) (marked as a check‐box variable or by ICD‐10 codes O24.4, O24.9).^19^ Severe birth injuries were defined according to Muraca et al. (2018),^2^ (Table 1). A composite outcome of any severe birth injury was defined as one or more of the injuries described above and was referred to as “severe birth injury”. Data concerning pre‐pregnancy body mass index (BMI, kg/m^2^) were included after 2006, as values from several hospitals were missing for the years 2004 and 2005. Birthweights above +2 standard deviation (SD) were defined as large for gestational age (LGA) standardized for parity, sex and gestational age in a Finnish population.^20^ The use of oxytocin was registered if it was used to induce and/or augment labor. Spontaneous vaginal deliveries (SVDs) included spontaneous and induced deliveries as opposed to VAD.

**TABLE 1:** The frequency and incidence of individual types of severe birth injury associated with different types of diabetes among singleton vaginal deliveries with cephalic presentation between 35^+0^ and 42^+6^ gestational weeks from 2004 to 2017 in Finland

Type of birth injury	ICD‐10 codes	T1D (*n *= 1659)	T2D (*n *= 548)	GDM (*n *= 77 810)	No diabetes (*n *= 543 632)	Total (*n *= 623 649)
Intracranial hemorrhage or laceration	P10–P10.9	1 (0.06)	1 (0.18)	17 (0.02)	59 (0.01)	78 (0.01)
Severe central nervous system injury	P11.0–P11.2, P11.4–P11.5	– (0.00)	– (0.00)	2 (0.003)	5 (0.001)	7 (0.001)
Subaponeurotic hemorrhage	P12.2	2 (0.12)	– (0.00)	19 (0.02)	119 (0.02)	140 (0.02)
Skull fracture, long bone injury / fracture^a^	P13.0, P13.2, P13.3	3 (0.18)	– (0.00)	12 (0.02)	42 (0.008)	57 (0.009)
Brachial plexus injury	P14.0–P14.3	36 (2.17)	9 (1.64)	372 (0.48)	1253 (0.23)	1670 (0.27)
Injury to the liver or spleen	P15.0, P15.1	– (0.00)	– (0.00)	– (0.00)	– (0.00)	– (0.00)
Severe birth injury^b^		42 (2.53)	10 (1.82)	415 (0.53)	1467 (0.27)	1934 (0.31)
Total		42 (2.53)	10 (1.82)	422 (0.54)	1478 (0.27)	1952 (0.31)

Note

Data presented as number (% of live births).

Abbreviations: GDM, gestational diabetes; T1D, Type 1 diabetes; T2D, Type 2 diabetes.

^a^ Not including clavicle fractures.

^b^ Composite outcome, one or more injuries described above.

Management of diabetic pregnancies are based on national guidelines^19^, ^21^ and is uniform throughout the country. Women with pregestational diabetes or GDM needing pharmacological treatment for glycemic control are regularly guided by physicians and midwifes specialized to treat diabetic pregnant women. According to guidelines delivery is recommended between 38 and 40 weeks of gestation for women with pregestational diabetes or GDM with pharmacological treatment, and before 41^+3^ for dietary treated GDM. The decision of the mode of delivery is based on the obstetrical indications if the estimated fetal weight by antenatal ultrasound is between 4000 and 4250 g in pregestational diabetes and up to 4500 g in medication treated GDM. Furthermore, an elective cesarean section is recommended if the estimated fetal weight is >4500 g in pregnancies with T1D, T2D and medication treated GDM.^19^, ^21^ Mediolateral episiotomy is performed only when deemed necessary. Birth injuries are primarily diagnosed by pediatric clinical examination. Radiologic evaluation is performed when severe birth injury is suspected and a specialized physician such as a pediatric surgeon is consulted.

### 2.1 Statistical analysis

The incidences of composite severe birth injury as well as individual types of injuries were calculated. Baseline characteristics were described as proportions for categorical variables and as means and SDs or medians with inter‐quartile ranges for continuous variables. The background characteristics in different diabetes categories were compared using chi squared‐test and Fisher's exact test for categorical variables and Welch Two Sample *t*‐test and Mann–Whitney *U*‐test for continuous variables. The risk factor analysis was calculated using a composite severe birth injury as an outcome variable. The results are presented as odds ratios (ORs), risk differences (RDs), and ratio of odds ratios (OR‐ratios; the ratio of odds ratios for a severe birth injury with a given risk factor in diabetic groups versus non‐diabetic group), with 95% confidence intervals (CIs). A logistic regression analysis was performed for the variables associated with the highest risk for injury. *P*‐value of <0.05 was considered significant. Statistical analysis was performed using R Statistical Software version 4.0.3 (R Foundation for Statistical Computing, Vienna, Austria).

### 2.2 Ethical approval

Only pseudonymized data were used. This study was approved by the Ethics committee of Tampere University Hospital (reference number R17069). Institutional approval was also obtained from the Finnish Institute for Health and Welfare (reference number THL/1659/5.05.00/2017).

## 3 RESULTS

The study population consisted of 623 649 neonates with 1952 severe birth injuries in 1934 neonates. The total incidence of injuries was 1952/623 649 (0.3%) of live births in vaginal deliveries (Table 1). The injury incidence was highest in women with T1D and T2D: 42/1659 (2.5%) and 10/548 (1.8%) of live births, respectively. BPP was the most frequent injury (*n *= 1670), accounting for 85.6% of all severe injuries. Other severe birth injuries were infrequent.

In diabetic pregnancies, labors were induced, oxytocin was used more often, and neonates were born earlier compared to non‐diabetic pregnancies. In pregnancies with T1D ShD, LGA, and VAD occurred most often, whereas the incidences of VAD and ShD were similar between women with GDM and non‐diabetic women (Table 2).

**TABLE 2:** Maternal and delivery characteristics by diabetes type in singleton vaginal delivery with cephalic presentation between 35^+0^ and 42^+6^ gestational weeks

	T1D	T2D	GDM	No diabetes
Live births	1659	548	77 810	543 632
Spontaneous vaginal delivery	1386 (83.5)	481 (87.8)	69 496 (89.3)	488 189 (89.8)
Vacuum‐assisted delivery	273 (16.5)	67 (12.2)	8314 (10.7)	55 443 (10.2)
Age (years)	29.7 ± 5.47	32.6 ± 5.68	31.0 ± 5.40	29.3 ± 5.26
BMI (kg/m^2^)	25.9 ± 5.87	31.8 ± 7.50	28.0 ± 6.00	23.6 ± 4.20
Smoking	279 (16.8)	130 (23.7)	14 454 (18.6)	92 028 (16.9)
Primiparity	635 (38.3)	174 (31.8)	27 141 (34.9)	219 207 (40.3)
Previous cesarean section	193 (11.6)	66 (12.0)	7147 (9.2)	36 180 (6.7)
Induction of labor	1056 (63.7)	339 (61.9)	25 145 (32.3)	97 826 (18.0)
Use of oxytocin	994 (59.9)	322 (58.8)	39 509 (50.8)	239 940 (44.1)
Epidural and/or spinal anesthesia	1123 (67.7)	359 (65.5)	49 229 (63.3)	325 482 (59.9)
Paracervical and/or pudendal block	409 (24.7)	141 (25.7)	19 982 (25.7)	126 000 (23.2)
Shoulder dystocia	60 (3.6)	5 (0.9)	477 (0.6)	1617 (0.3)
Episiotomy	499 (30.1)	93 (17.0)	17 783 (22.9)	134 882 (24.8)
LGA	421 (25.4)	55 (10.0)	2835 (3.6)	8203 (1.5)
Birthweight (grams)	3709.8 ± 494.52	3599.4 ± 471.04	3634.6 ± 479.86	3552.4 ± 463.86
Gestational age (weeks^+days^)	38^+1^ (37^+1^–39^+0^)	38^+6^ (38^+1^–39^+6^)	39^+6^ (39^+0^–40^+4^)	40^+1^ (39^+2^–41^+0^)
Infant sex (boys)	775 (46.7)	289 (52.7)	39 839 (51.2)	274 259 (50.5)

Note

Data presented *n* (% of vaginal live births), mean ± SD or median with inter‐quartile range.

Abbreviations: BMI, body mass index; GDM, gestational diabetes; LGA, large for gestational age; T1D, Type 1 diabetes; T2D, type 2 diabetes.

The strongest risk factors for severe birth injury were ShD, LGA, and VAD in all study groups (Table 3). The highest risk for injury was associated with ShD in all women. (T1D: OR 24.89, 95% CI 12.53–49.46, T2D: OR 114.86, 95% CI 16.53–797.94, GDM: OR 82.79, 95% CI 65.25–105.03, non‐diabetic: OR 106.62, 95% CI 92.91–122.35). One‐third of all neonates who experienced ShD had a severe birth injury. Based on OR‐ratio, ShD was a more powerful risk factor for non‐diabetic women compared to women with T1D, and a similar tendency was observed in pregnancies with GDM. The incidence of injuries among LGA newborns ranged between 214/8203 (2.6%) and 28/421 (6.7%), being highest in women with T1D. In total, from 505/55 443 (0.9%) to 6/67 (9.0%) of VADs resulted in injury, with the highest incidences in the T1D and T2D groups. Considering RDs, LGA and VAD only moderately increased the risk for injury. Primiparity and smoking were moderate risk factors for injury in women with T1D. Labor induction, use of oxytocin, and epidural or spinal anesthesia were associated with an increased risk for injury in women with GDM and non‐diabetic women. Based on the RD, the increased probability of injury associated with these factors was, however, quite low (0.07%–0.34%, 95% CI 0.04–0.10% and 0.23–0.44%).

**TABLE 3:** Risk factors for neonatal severe birth injury by diabetes type in singleton vaginal delivery with cephalic presentation between 35^+0^ and 42^+6^ gestational weeks

	T1D	T2D	GDM	No diabetes
Number of live births	1659	548	77 810	543 632
Injured neonates (n)	42	10	415	1467
Shoulder dystocia (n)	60	5	477	1617
Injured neonates of total no. of neonates with risk factor^a^	17 (28.3)	3 (60.0)	116 (24.3)	302 (18.7)
Injured neonates with risk factor of all injured neonates^a^	17 (40.5)	3 (30.0)	116 (28.0)	302 (20.6)
OR (95% CI)^b^	24.89 (12.53–49.46)	114.86 (16.53–797.94)	82.79 (65.25–105.03)	106.62 (92.91–122.35)
*P* ^c^	<0.001	<0.001	<0.001	<0.001
RD (95% CI)^d^	26.77 (16.92–39.21)	58.71 (21.76–86.96)	23.93 (20.30–27.98)	18.46 (16.64–20.43)
OR‐ratio (95% CI)^e^	0.23 (0.12–0.47)	1.08 (0.15–7.52)	0.78 (0.59–1.02)	
*P* (OR)^f^	<0.001	0.940	0.071	
LGA (n)	421	55	2853	8203
Injured neonates of total no. of neonates with risk factor^a^	28 (6.7)	2 (3.6)	110 (3.9)	214 (2.6)
Injured neonates with risk factor of all injured neonates^a^	28 (66.7)	2 (20.0)	110 (26.5)	214 (14.6)
OR (95% CI)^b^	6.23 (3.25–11.95)	2.29 (0.47–11.05)	9.88 (7.92–12.33)	11.42 (9.86–13.22)
*P* ^c^	<0.001	0.303	<0.001	<0.001
RD (95% CI)^d^	5.52 (3.37–8.35)	2.01 (−1.04 to 10.74)	3.47 (2.82–4.25)	2.37 (2.05–2.74)
OR‐ratio (95% CI)^e^	0.55 (0.28–1.06)	0.20 (0.04–0.97)	0.87 (0.66–1.13)	
*P* (OR)^f^	0.075	0.046	0.286	
Vacuum‐assisted delivery (n)	279	67	8314	55 443
Injured neonates of total no. of neonates with risk factor^a^	15 (5.5)	6 (9.0)	155 (1.9)	505 (0.9)
Injured neonates with risk factor of all injured neonates^a^	15 (35.7)	6 (60.0)	155 (37.4)	505 (34.4)
OR (95% CI)^b^	2.93 (1.54–5.58)	11.73 (3.22–42.74)	5.06 (4.14–6.18)	4.66 (4.18–5.19)
*P* ^c^	0.001	<0.001	<0.001	<0.001
RD (95% CI)^d^	3.55 (1.24–6.97)	8.12 (3.17–17.38)	1.49 (1.22–1.81)	0.71 (0.64–0.80)
OR‐ratio (95% CI)^e^	0.63 (0.33–1.21)	2.52 (0.69–9.22)	1.09 (0.87–1.36)	
*P* (OR)^f^	0.164	0.162	0.474	
Primiparity (n)	635	174	27 141	219 207
Injured neonates of total no. of neonates with risk factor^a^	23 (3.62)	4 (2.3)	156 (0.6)	622 (0.3)
Injured neonates with risk factor of all injured neonates^a^	23 (54.8)	4 (40.0)	156 (37.6)	622 (42.4)
OR (95% CI)^b^	1.99 (1.07–3.68)	1.44 (0.40–5.18)	1.13 (0.92–1.37)	1.09 (0.98–1.21)
*P* ^c^	0.029	0.733	0.256	0.105
RD (95% CI)^d^	1.77 (0.19–3.64)	0.69 (−1.62 to 4.26)	0.06 (−0.04 to 0.18)	0.02 (−0.005 to 0.05)
OR‐ratio (95% CI)^e^	1.82 (0.98–3.41)	1.32 (0.37–4.77)	1.03 (0.82–1.29)	
*P* (OR)^f^	0.059	0.668	0.780	
Smoking (n)	279	130	14 454	92 028
Injured neonates of total no. of neonates with risk factor^a^	13 (4.7)	2 (1.5)	84 (0.6)	228 (0.3)
Injured neonates with risk factor of all injured neonates^a^	13 (31.0)	2 (20.0)	84 (20.2)	228 (15.5)
OR (95% CI)^b^	2.28 (1.17–4.43)	0.80 (0.17–3.82)	1.11 (0.88–1.42)	0.90 (0.78–1.04)
*P* ^c^	0.016	0.780	0.382	0.160
RD (95% CI)^d^	2.56 (0.44–5.77)	−0.38 (−2.51 to 3.63)	0.06 (−0.07 to 0.21)	−0.03 (−0.06 to 0.01)
OR‐ratio (95% CI)^e^	2.52 (1.27–4.98)	0.89 (0.18–4.25)	1.23 (0.93–1.63)	
*P* (OR)^f^	0.008	0.880	0.142	
Labor induction (n)	1056	339	25 145	97 826
Injured neonates of total no. of neonates with risk factor^a^	31 (2.9)	7 (2.1)	172 (0.7)	352 (0.4)
Injured neonates with risk factor of all injured neonates^a^	31 (73.8)	7 (70.0)	172 (41.5)	352 (24.0)
OR (95% CI)^b^	1.63 (0.81–3.26)	1.45 (0.37–5.66)	1.49 (1.22–1.81)	1.44 (1.28–1.62)
*P* ^c^	0.170	0.595	<0.001	<0.001
RD (95% CI)^d^	1.11 (−0.54 to 2.56)	0.63 (−2.27 to 2.97)	0.22 (0.11–0.35)	0.11 (0.07–0.15)
OR‐ratio (95% CI)^e^	1.13 (0.56–2.29)	1.01 (0.26–3.95)	1.03 (0.82–1.30)	
*P* (OR)^f^	0.734	0.994	0.790	
Use of oxytocin (n)	994	322	39 509	239 940
Injured neonates of total no. of neonates with risk factor^a^	32 (3.2)	5 (1.6)	276 (0.7)	805 (0.3)
Injured neonates with risk factor of all injured neonates^a^	32 (76.2)	5 (50)	276 (66.5)	805 (54.9)
OR (95% CI)^b^	2.18 (1.06–4.46)	0.70 (0.20–2.44)	1.93 (1.57–2.37)	1.54 (1.39–1.71)
*P* ^c^	0.033	0.572	<0.001	<0.001
RD (95% CI)^d^	1.72 (0.16–3.18)	−0.66 (−3.66 to 1.73)	0.34 (0.23–0.44)	0.12 (0.09–0.15)
OR‐ratio (95% CI)^e^	1.41 (0.69–2.92)	0.45 (0.13–1.59)	1.25 (1.0–1.58)	
*P* (OR)^f^	0.349	0.216	0.053	
Epidural and/or spinal anesthesia (n)	1123	359	49 229	325 482
Injured neonates of total no. of neonates with risk factor^a^	35 (3.1)	7 (1.9)	293 (0.6)	973 (0.3)
Injured neonates with risk factor of all injured neonates^a^	35 (83.3)	7 (70.0)	293 (70.6)	973 (66.3)
OR (95% CI)^b^	2.43 (1.07–5.51)	1.23 (0.32–4.82)	1.40 (1.13–1.73)	1.32 (1.19–1.47)
*P* ^c^	0.033	>0.99	0.002	<0.001
RD (95% CI)^d^	1.81 (0.19–3.17)	0.36 (−2.78 to 2.64)	0.17 (0.06–0.27)	0.07 (0.04–0.10)
OR‐ratio (95% CI)^e^	1.84 (0.81–4.20)	0.93 (0.24–3.67)	1.06 (0.83–1.34)	
*P* (OR)^f^	0.148	0.921	0.647	

^a^ Values are given as number (percentage).

^b^ OR; Odds ratio representing the odds for a severe birth injury in the T1D, T2D, GDM, or non‐diabetes groups with a given risk factor versus women without a risk factor.

^c^ P‐value calculated from incidence rate ratio, using Chi‐square and Fisher's exact test.

^d^ RD; Risk difference representing the difference between the risk for a severe birth injury in the group exposed to risk factor versus the group unexposed to risk factor. Values are presented as absolute numbers × 100.

^e^ OR‐ratio; The ratio of Odds Ratios for a severe birth injury in the diabetes group (T1D, T2D or GDM) versus the non‐diabetic group within a given risk factor. OR‐ratio >1 meaning a higher Odds Ratio in the group of women with diabetes versus a group of non‐diabetic women.

^f^ P (OR); P‐value from OR‐ratio, based on calculated log‐OR difference, standard error, and Wald test statistic (Z score).

**FIGURE 1:**
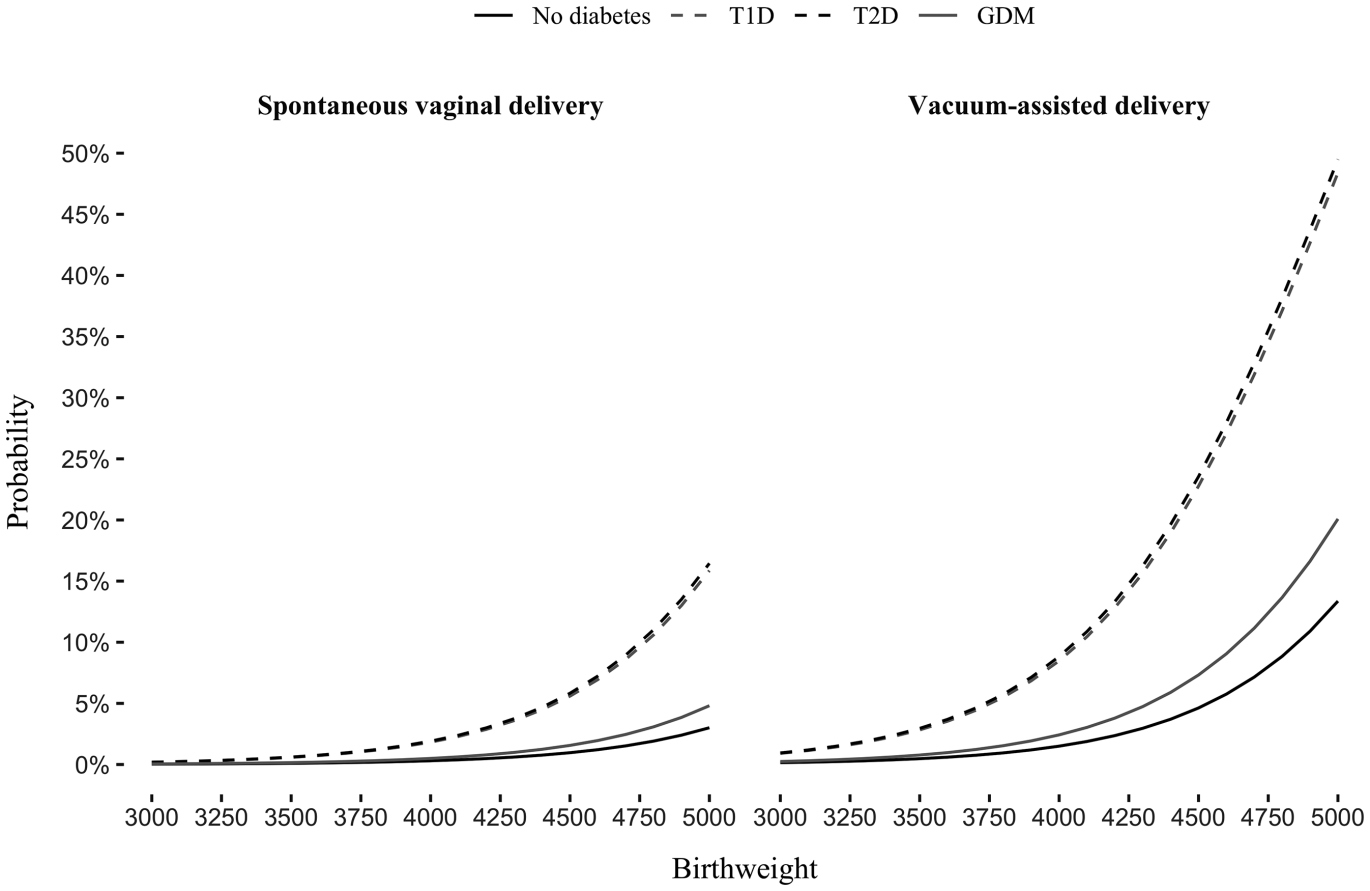
The probability of severe birth injury in relation to birthweight, diabetes type, and mode of delivery

The probability of injury after SVD in pregnancies with T1D or T2D began to increase with a birthweight of more than 3900 g and more steeply with a birthweight of more than 4300 g. The risk was further increased in VAD (Figure 1, Table 4). However, for neonates born by SVD to women with GDM or non‐diabetic women, the probability of injury remained low up to a birthweight of 4500 g. The effect of high birthweight on injury probability was clearly seen among neonates born by VAD to women with GDM or to non‐diabetic women with a birthweight of more than 4300 g. High birthweight per se was a more important risk factor for injury than LGA (LGA’s regression curve not shown).

**TABLE 4:** The probability of neonatal severe birth injury by diabetes type in singleton vaginal delivery with cephalic presentation between 35^+0^ and 42^+6^ gestational weeks

	Probability of severe birth injury in vaginal delivery % (95% CI)			
	T1D	T2D	GDM	No‐diabetes
Birthweight 3000 g				
SVD	0.18 (0.13–0.26)	0.19 (0.10–0.37)	0.05 (0.04–0.06)	0.03 (0.03–0.03)
VAD	0.91 (0.65–1.27)	0.95 (0.50–1.80)	0.24 (0.21–0.29)	0.15 (0.13–0.17)
Birthweight 3500 g				
SVD	0.58 (0.42–0.80)	0.61 (0.32–1.15)	0.16 (0.14–0.18)	0.10 (0.09–0.10)
VAD	2.83 (2.05–3.89)	2.95 (1.57–5.47)	0.77 (0.68–0.89)	0.48 (0.43–0.52)
Birthweight 4000 g				
SVD	1.83 (1.33–2.50)	1.91 (1.01–3.56)	0.50 (0.45–0.55)	0.31 (0.29–0.32)
VAD	8.48 (6.29–11.34)	8.83 (4.85–15.54)	2.42 (2.22–2.71)	1.50 (1.39–1.62)
Birthweight 4500 g				
SVD	5.59 (4.12–7.55)	5.83 (3.15–10.52)	1.56 (1.40–1.74)	0.96 (0.90–1.04)
VAD	22.77 (17.56–28.97)	23.55 (13.92–36.96)	7.32 (6.53–8.20)	4.62 (4.23–5.05)
Birthweight 5000 g				
SVD	15.86 (11.94–20.75)	16.45 (9.35–27.32)	4.81 (4.24–5.44)	3.01 (2.71–3.34)
VAD	48.40 (40.20–56.69)	49.49 (33.86–65.23)	20.09 (17.86–22.51)	13.36 (12.0–14.56)

Abbreviations: GDM, gestational diabetes; SVD, spontaneous vaginal delivery; T1D, Type 1 diabetes; T2D, Type 2 diabetes; VAD, vacuum‐assisted delivery.

**FIGURE 2:**
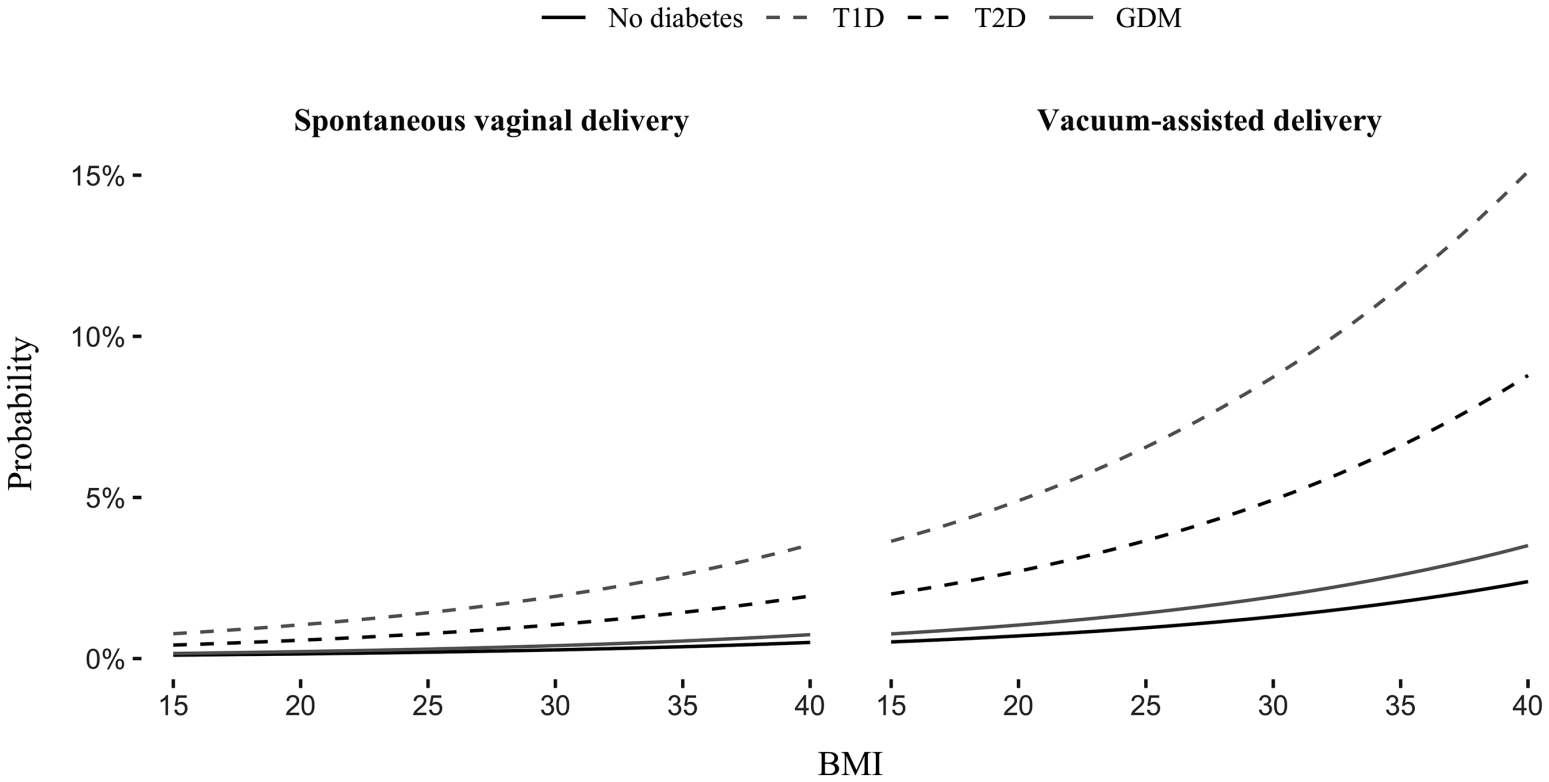
The probability of severe birth injury in relation to BMI, diabetes type, and mode of delivery

A high BMI was associated with a risk for birth injury, but BMI had a lower impact than birthweight. Obesity was associated with the risk for injury in VAD in women with pregestational diabetes, but the probability of injury remained low in SVD in women with GDM and non‐diabetic women, even in those with severe obesity (Figures 1 and 2). In logistic regression analysis, shorter maternal height, older maternal age, neonate's higher birth length, and higher gestational age were independently associated with increased risk for injury in all groups studied, but the impact of above‐mentioned risk factors was not clinically relevant (data not shown).

## 4 DISCUSSION

In this population‐based study, the incidence of severe birth injury was highest in pregnancies complicated by T1D and T2D. Severe birth injuries, other than BPP, were infrequent. Risk factors were similar in diabetic and non‐diabetic women, but high birthweight and obesity had a stronger impact on severe birth injuries in women with pregestational diabetes compared to GDM and non‐diabetic pregnancies. This impact was further increased by vacuum extraction. The risk for injury in neonates delivered by women with GDM or non‐diabetic women was generally low when labors complicated by ShD and the VAD of high birthweight neonates were excluded.

The distribution of severe birth injury, other than BPP, by different types of maternal diabetes has not previously been reported. The incidence of BPP among the neonates of diabetic women was similar (T1D: 2.2% of vaginal live births, GDM: 0.6% of vaginal live births),^7^, ^11^ and the total incidence of BPP was higher than previously described in the literature (0.11% to 0.16% of vaginal births).^2^, ^23^ ShD was the strongest risk factor associated with injury in all neonates irrespective of the diabetes status of the parturient, which is in line with the findings of previous studies.^6^, ^22^ Furthermore, the incidence of ShD was similar to that previously reported.^9^, ^22^ Regarding BPP, however, the rates of injured neonates after ShD were higher than those reported in previous studies.^22^ The high comorbidity in the present study may be due to diverse diagnostic criteria or the broad coverage of the data, as we included all severe birth‐related injuries diagnosed during the first year after birth. Moreover, we cannot rule out the possibility of underdiagnosing the milder forms of ShD without birth injury. The overall incidence of severe birth injury and the incidences of subaponeurotic hemorrhage and intracranial hemorrhage were comparable to those reported in previous studies (0.2%, 0.01%, and 0.02% of vaginal births, respectively).^2^

Baseline characteristics differed considerably between the types of diabetes, as reported earlier by others.^4^, ^5^, ^8^ T1D pregnancies had the highest incidences of the main risk factors, namely LGA, ShD, and VAD, explaining the high injury rate. Women with T2D and GDM had a higher BMI, and neonates were more often LGA compared with the neonates of non‐diabetic women. Obesity, along with GDM and maternal pregestational diabetes, have been suggested to be independent risk factors for BPP.^6^, ^7^ In this study, high BMI increased the risk for injury in neonates born by VAD in women with pregestational diabetes, but it was a less important risk factor in women with GDM and in non‐diabetic women.

High birthweight was the most important risk factor for birth injury. Although ShD is unpredictable, it is often associated with high birthweight, and high birthweight is suggested to be an independent risk factor for birth injury among neonates with ShD.^6^, ^10^, ^11^ In line with a previous publication,^11^ the birthweight per se was a more important risk factor for injury than LGA. The importance of birthweight as a risk factor for birth injury was most clearly seen in T1D and T2D pregnancies and further strengthened by VAD. On the other hand, the probability of injury was almost the same when comparing the pregnancies of women with GDM and non‐diabetic women and remained rather low with higher birthweights among SVD. Nevertheless, the probability of injury also began to rise with birthweights above 4000 g in the neonates of women with GDM, if VAD was required. This increased risk for injury in neonates born by VAD, especially those with high birthweight, is in concordance with previous reports.^6^, ^22^ Approximately one‐third of the injured neonates were born by vacuum extraction. Thus, promoting SVD may be one way to reduce the rate of birth injuries. The predictability of the risk of injury based on birthweight was less consistent in the neonates of women with GDM or in non‐diabetic women than it was in women with pregestational diabetes. Perhaps because of the low incidences of ShD and LGA, the injuries among the neonates of non‐diabetic women occurred less often concomitant with ShD or LGA than injuries associated with maternal diabetes. A similar relationship was also reported by Johnson et al.^23^

There is no standardized screening system or criteria for GDM. Indeed, it has been recently questioned whether the comprehensive screening of GDM and the treatment of mild hyperglycemia are worthwhile, and is the current system only increasing the number of women with GDM without improvement in outcomes.^16^, ^24^ Nevertheless, it has been shown that there is a linear association between hyperglycemia and adverse pregnancy outcomes, and an association between mild untreated hyperglycemia and higher birthweight. Moreover, the treatment of GDM at least decreases the risk for ShD and high birthweight.^4^, ^25^ In this study, the incidence of birth injury was comparable in the neonates of women with GDM and in non‐diabetic women, suggesting that without screening and treatment the incidence may well have been higher.

The strengths of the present study are the statutory Finnish MBR and CRHC data with national coverage ruling out selection bias and increasing generalizability. The precision and completeness of the data have been reported to be good.^17^, ^18^ In Finland, maternal and child welfare clinics are free of charge, ensuring equal opportunity for care and attendance by the entire pregnant population. The limitation of the study is the retrospective nature of the data. Moreover, the diagnostic criteria for GDM changed during the study period. Even with a large sample size, the number of T2D pregnancies remained modest, limiting the statistical power of the results. BPP, as the most common injury, influenced the results, and therefore the risk factors represent primarily risk factors for BPP.

## 5 CONCLUSION

The neonates of women with pregestational diabetes have a higher risk for severe birth injury than other neonates. The risk is strongly associated with ShD, higher birthweight and further strengthened by VAD. The incidence of injury in pregnancies with GDM is comparable with pregnancies without diabetes. Moreover, the impact of high birthweight and obesity on the risk for injury in GDM and non‐diabetic pregnancies is less important than in women with pregestational diabetes.

## CONFLICTS OF INTEREST

The authors have no conflicts of interest.

## AUTHOR CONTRIBUTIONS

MK, TH, KT, HL, and AS designed the study. TH, AS, and MG contributed to the acquisition of the data. MK, TH, and TK were responsible for data analysis. All authors contributed to the interpretation of the data. MK was a major contributor in the writing of the manuscript. All authors participated in drafting and revising the manuscript. All authors read and approved the final manuscript.

## Data Availability

The data that support the findings of this study are available on request from the corresponding author.
